# Smoking Cessation and the Internet: A Qualitative Method Examining Online Consumer Behavior

**DOI:** 10.2196/jmir.4.2.e8

**Published:** 2002-11-22

**Authors:** Genevieve Frisby, Tracey L Bessell, Ron Borland, Jeremy N Anderson

**Affiliations:** ^1^Monash Institute of Health Services ResearchMelbourneAustralia; ^2^Cancer Councel of VictoriaVicHealth Center for Tobacco ControlAustralia; ^3^Southern Health/Monash Institute of Health Services ResearchCentre for Clinical EffectivenessMelbourneAustralia

**Keywords:** smoking cessation, Internet, health behavior, health promotion

## Abstract

**Background:**

Smoking is a major preventable cause of disease and disability around the world. Smoking cessation support — including information, discussion groups, cognitive behavioral treatment, and self-help materials — can be delivered via the Internet. There is limited information about the reasons and methods consumers access smoking cessation information on the Internet.

**Objectives:**

This study aims to determine the feasibility of a method to examine the online behavior of consumers seeking smoking cessation resources. In particular, we sought to identify the reasons and methods consumers use to access and assess the quality of these resources.

**Methods:**

Thirteen participants were recruited via the state-based Quit® smoking cessation campaign, operated by the Victorian Cancer Council, in December 2001. Online behavior was evaluated using semi-structured interviews and Internet simulations where participants sought smoking cessation information and addressed set-case scenarios. Online interaction was tracked through pervasive logging with specialist software.

**Results:**

Thirteen semi-structured interviews and 4 Internet simulations were conducted in January 2002. Participants sought online smoking cessation resources for reasons of convenience, timeliness, and anonymity — and because their current information needs were unmet. They employed simple search strategies and could not always find information in an efficient manner. Participants employed several different strategies to assess the quality of online health resources.

**Conclusions:**

Consumer online behavior can be studied using a combination of survey, observation, and online surveillance. However, further qualitative and observational research is required to harness the full potential of the Internet to deliver public health resources.

## Introduction

"Every eight seconds a person dies of a tobacco-related disease and almost as quickly another victim is recruited." [1]

The World Health Organization (WHO) attributes about 4 million deaths a year to tobacco use, a figure expected to rise to about 10 million deaths a year by 2030 [2]. Smoking kills 19000 Australians each year and represents the major preventable cause of disease and disability in Australia [3]. Tobacco is highly addictive and quitting smoking is the single most important action a smoker can take to improve his or her health [4].

Smoking cessation information and supportive resources such as discussion groups, cognitive behavioral treatments, and tailored self-help materials can be delivered via the Internet. Nicotine-replacement therapies can also be purchased online. In May 2002, it was estimated that almost 10% (580 million) of the global population have accessed the Internet [5]. In Australia, 50% of Australian adults and 37% of households have Internet access and more than 25% of Australians aged between 15 and 54 years of age seek online health resources [6- 7]. Thus the Internet can potentially deliver smoking cessation interventions, resources, and information to a critical mass of consumers.

There are many Web sites concerned with smoking cessation. (For example, using the Google search engine and the terms *stop* *smoking* returned 940000 hits and *smoking* *cessation* returned 247000 hits on February 5, 2002.) Despite the volume of online information, we could not identify any published studies about the quality of online information about self-help smoking cessation. The overall quality of online health information is variable, sometimes fraudulent and misleading [8]. We lack understanding about whether consumers can differentiate between good and poor quality Web sites. Further, we do not know why consumers access online smoking cessation resources in preference to telephone helplines or face-to-face health-professional advice.

This study determines the feasibility of a method to examine the online behavior of consumers seeking smoking cessation information. In particular, we aim to identify the reasons consumers choose to use the Internet to obtain smoking cessation information and how they conduct searches for online information. The results of this project will inform those interested in realizing the full potential of the Internet to deliver smoking cessation interventions and resources to the broader community in a timely, convenient, and economical manner.

## Methods

The Victorian Quit® program is the major state agency providing a range of supports to smokers wanting to quit and to health professionals whose roles include encouraging smoking cessation. It produces a range of written materials, most reproduced on its Web site, and has an extensive statewide telephone service. Callers to that service are offered a set of printed cessation resources and/or access to trained cessation counsellors. We recruited a convenience sample of participants for this study from those consumers contacting the telephone helpline who agreed to be contacted for research purposes and via an advertisement placed on the state-based Quit® Web site operated by The Cancer Council Victoria, Australia [9]. We invited consumers to participate in a semi-structured interview and an online simulation regarding use of the Internet for smoking cessation purposes.

We reimbursed participants Aus $40 to cover their costs and an additional Aus $20 if they participated in the Internet simulations. All participants gave informed signed consent and were free to withdraw from the study at any time. Ethics approval was granted from the relevant Southern Health Human Research Ethics Committee, Monash Medical Centre.

We recruited 13 participants in early January 2002. By chance, we recruited all participants via the Quit® Web site and all participants were currently trying to stop smoking. We conducted 13 semi-structured interviews involving 4 males and 9 females, aged 19 to 64 years (median 30-39 years). Twelve participants used the Internet at least once a week and had Internet access at work or home. The amount of time spent online ranged from 30 minutes to 10-12 hours per week. Nine of the 13 participants had obtained a Bachelor of Higher Degree. Two participants lived in non-metropolitan locations. Two participants had total household incomes less than the Australian average gross household income (which is slightly lower than Aus $40000) [10]. Four of the 13 participants voluntarily chose to participate in the Internet simulations.

We conducted semi-structured interviews (Appendix 1) by telephone or face-to-face, audio-taped, then transcribed. We employed qualitative research methods to collect context-bound data that help to predict, explain, or understand a particular phenomenon. GF employed a framework approach to analyze the interview data (Table 1) [11]. This approach starts deductively(reasoning from general to particular instances) from the aims and objectives set for the study and the results are grounded (heavily based in the original accounts and observations of the people studied) and inductive (employing the process of inferring a principle from the observation of particular instances). Such an approach is advantageous because the analytic process and interpretations can be viewed and assessed by people other than the primary analyst. TB reviewed the resultant themes and conflicting views. Themes were identified using a constant comparison method, whereby each category is searched in the entire data set and all instances are compared until no new categories emerge; a time intensive process.

**Table 1:** The 5 stages of data analysis using a framework approach

1. Familiarization -- immersion in the raw data by reading transcripts, in order to list key ideas and recurrent themes
2. Identifying a thematic framework -- identify all the key issues, concepts and themes by which the data can be examined and referenced by drawing on prior knowledge, the aims and objectives of the study, and issues raised by participants
3. Indexing -- applying the thematic framework to all the data using codes
4. Charting -- rearranging the data according to themes. The end result is a chart for each key theme containing distilled summaries of participants' views and experiences
5. Mapping and interpretation -- using the charts to define concepts, map the range and nature of the phenomena, create typologies, and find associations between themes with a view to providing explanations for the findings

We used a desktop computer with Internet access to conduct the simulations. We initially asked participants to seek general online smoking cessation information and secondly asked participants to address 3 set-case scenarios (Appendix 2). We tracked participants' online interaction through pervasive logging with specialized software (Omniquad Desktop Surveillance Personal Edition ® [12]). This software records the search engines and terms used and the sites visited. It also makes a screen capture every minute, so that the log can be cross-referenced to ensure that the correct site information is recorded. The simulations were observed by a research assistant who also noted the search engines, key phrases, Web sites visited, and any verbal comments made by participants.

## Results

### General information

Participants most commonly perceived that people found out about ways to stop smoking via television and family or friends. However, they had all sought online smoking cessation information for reasons of convenience. Based upon past personal experiences the participants also felt that there were benefits of being able to access health information — especially smoking cessation information — anonymously; some felt uncomfortable speaking to health professionals about quitting.

For example, one participant said, " *You don't have to speak to people who make you feel bad.*"

Five participants felt that online health information was preferable to other information sources because it offered a global perspective and the opportunity to find specific information.

All participants self-reported using commercial search engines and none accessed health-specific search engines or portals during the Internet simulations. A variety of search terms were employed, most commonly *quit* *smoking*.

Participants sought and found information about many themes upon seeking general information about smoking cessation (Table 2,Table 3).

**Table 2:** Types of information sought by consumers* (N=13)

Category	Number of responses
Support available	3
General information	3
Weight gain when quitting	2
Support you can offer others	2
Strategies for stopping	2
About Quit REG_ENTITY	1
Nicotine Replacement Therapy	1
Products	1
Dealing with cravings	1
Coping with stress when quitting	1
**Total**	17

**Table 3:** Types of information found by consumers* (N = 13)

Category	Number of responses
Stop smoking advice/strategies	7
Support services	5
Sites selling products for quitting	5
Nicotine Replacement Therapy	3
Benefits of stopping	2
Diet plan for quitters	1
Descriptive statistics on prevalence of smoking and related adverse health outcomes	1
Reasons to stop smoking	1
Quit REG_ENTITY course information	1
Personalized calendar	1
Sites selling cigarettes or tobacco products	1
Stress management information	1
**Total**	29

^*^ Participants could give multiple responses.

Nine of the 13 participants stated that general information about quitting was valuable. In particular, quitting strategies, the benefits of quitting, practical information about weight gain, and stress management when quitting were useful. Six of the 13 participants identified the Australian National Tobacco Campaign Web site [13] as the most useful. The Quit Book® was highlighted as a good source of information. The Quit Book® is an Internet version of an information booklet distributed as the main written resource to callers to the Quitline® telephone advisory service. It provides a staged approach to quitting and provides tips and other advice.

Participants responded positively to the interactive and personalized elements, such as online questionnaires or "quizzes" where they could enter personal information and receive tailored information in response. For example, men would not receive information about smoking in pregnancy. Currently most written health information is "one size fits all." Three of the 13 participants self-reported that this tailored information was valuable.

One participant said, " *You can lose a lot of personal touch by quitting on-line - I looked for sites with a personal touch.*"

Some of the participants also highlighted interactive sites as being useful. In particular, items like quitting guides, calendars, chat rooms, online peer support, and money-saver and years-added-to-life calculators were identified as being valuable.

Most of the participants recommended using the Internet in combination with other support resources including health professionals.

One participant said, " *The internet is a good starter, but you can't ask questions.*"

Accessing alternative support was perceived as an opportunity to cross-check the reliability of the information available on the Internet.

Many participants perceived that the process of determining the reliability or quality of information was difficult and one participant indicated that it was a "very scary" prospect. Despite these uncertainties a range of tactics were identified (Table 4).

Overall, consumers felt that using government Web sites or Web sites associated with a known specialist or reputable organization were the best way to access reliable information. None of the participants checked the about-us or terms-and-conditions information published on Web sites.

**Table 4:** How consumers assess the reliability of Web sites when seeking smoking cessation information and support* (N = 13)

Consumer assessment	Number of responses
Government sites	6
Web sites associated with known organizations	5
If selling something, suspect site	5
References published	5
Rely on my judgement/common sense	2
Follow friend's recommendations	2
Compare information across sites	1
Cross-check with health professional	1
Visual quality of the Web site	1
Currency of information	1
Medical site	1
Secure site	1
**Total**	31

^*^ Participants could give multiple responses.

The least-valued items included descriptive statistics about the prevalence of smoking and adverse health effects, repetitive information, and the reasons for stopping smoking.

One participant said, " *We already know that... we hear the information about the damage caused by smoking all the time.*"

One participant searching for information about stress management felt that there were a lot of sites that made the link between smoking cessation and stress, but also felt that there was limited practical information about how to manage the stress.

All but one participant stated that they would recommend people seek smoking cessation information via the Internet. The dissenter would not recommend the Internet, because "there was a lot out there, but they aren't identified in the searches" and furthermore "Australian sites were too difficult to find."

Participants self-reported that the information they found on the Internet prompted them to modify their diet, and to consult health professionals and the state-based Quit® organization. They also self-reported sharing this information with others.

### Case scenarios

In the first-case scenario, upon trying to find information about nicotine-replacement therapies and whether patches are more effective than gum, 3 of the 4 participants identified a range of nicotine-replacement-therapy formulations including gum, patches, nasal sprays, and inhalators. The fourth participant was only able to identify gum and patches. None could make judgements about the comparative effectiveness of the formulation types based upon the information published on the 11 Web sites visited. Furthermore, despite the participants expressed desire to seek unbiased information about nicotine replacement therapies, only half the Web sites visited were government or independent sites. The remainder were commercial Web sites selling pharmaceuticals.

In the second-case scenario, overall, participants found it difficult to locate local support services despite the use of multiple search terms. Two participants said that they had found an international Web site that provided users with the opportunity to find their local support services, by entering their postcode. However, they were unable to find this feature on an Australian Web site.

In the third-case scenario, all participants identified that Zyban® (bupropion HCl) was available as a prescription-only medicine and could list the risks or side effects associated with use by accessing information published on the Internet. The manufacturer's Web site was accessed by all. Other Web sites accessed included current-affairs and consumer-affairs Web sites, Quit®, and Web sites selling pharmaceuticals.

## Discussion

Participants sought online smoking-cessation resources because they were convenient, timely, and anonymous. However, the quality of online health information is variable and the ranking of Web sites on commercial search engines is often influenced by money, not quality [14]. Despite these issues, consumers value online information and resources but cannot always find the information they need in a timely efficient manner, partly because they utilize simple search strategies and commercial search engines. However, these consumers were unsure how to assess the quality of online health information.

Our results are strikingly similar and directly support those of a recently-published paper examining how German consumers search for and appraise general health information on the Web [15]. However, our study is limited to an examination of the views of a small group of online health seekers who accessed the Quit® Web site and therefore may not reflect the experiences of the wider community of Australian smokers. Despite this limitation, the study demonstrated that the methodological design was feasible and these results provide a useful starting point to inform future research examining online consumer behavior. We intend to recruit a larger number of consumers for future studies using advertisements placed in Australian metropolitan and rural newspapers, in addition to the Quit® Web site and information packs.

Tobacco is highly addictive and many users are unable to voluntarily cease use, even when aware of the harm tobacco causes. A comparison of the information sought and that found by participants demonstrates that participants were able to access smoking cessation strategies and support services from around the world. However, those participants seeking complementary local resources such as face-to-face support groups or telephone counselling had difficulty locating Australian Web sites to access this information. Thus other types of media such as radio, television, and newspapers are necessary to promote local smoking cessation activities.

Approximately 80% of smokers in Australia have tried to quit at least once [16]. Effective relapse-prevention strategies are of utmost importance, given that most cessation attempts are unsuccessful [17]. Self-help interventions for smoking cessation suggest a modest effect [18] and there is increasing evidence on the effectiveness of computer and Internet mediated systems for the delivery of such interventions [19-21]. This study supports the further development of interactive and personalized smoking cessation tools delivered via the Internet because it demonstrates that consumers control and focus their use of the Internet to areas in which they are interested and need support/information. A randomized controlled trial examining the effectiveness of computer-tailored advice for smoking cessation and relapse prevention is currently being conducted in Australia by Borland R, Balmford J and Hunt D.

### Conclusions

Qualitative research can give rise to insights into emerging research areas by allowing us to collect context-bound data. This research demonstrates that the behavior of consumers seeking online health information and resources can be studied using a combination of survey, observation, and online surveillance. However, further qualitative and observational research regarding the online behavior of consumers is required if we are to harness the full potential of the Internet to deliver public health — in particular smoking cessation, interventions, and resources — to society.

## Appendix 1

### Semi-structured interview questions

First of all, I'm just going to start with some general questions about your use of the Internet...

- 1. How often do you use the Internet?
- 2. Where do you use the Internet? (e.g. library/home/work)
- 3. Approximately how much time do you spend per week (or month) on the Internet?
- 4. Have you ever searched for health information for other people (e.g. family/friends)?

The next questions relate more to stopping smoking...

- 5. In general, how do people find out about ways to stop smoking and the support services available?
- 6. Have you used the Internet to find out information about stopping smoking and the services available?
- 7. Given there are other ways of finding out about stopping smoking, why do you think people choose to use the Internet to obtain information about quitting?

The next questions are about specific stop smoking information you found on the Internet...

- 8. Before you started searching on the Internet, did you have anything that you wanted to know specifically? Were you expecting to find anything in particular?
- 9. How did you actually search for information about stopping smoking?
- 10. What type of information did you find?
- 11. How do you tell if information on the Internet is reliable?

Next, we are going to discuss the quality of the websites and what happened next for you...

- 12. What did you do with the information that you found?
- 13. How did you use this information?
- 14. What was the most useful/valuable information/item you found on the Internet about stopping smoking? Why was it so valuable?
- 15. What was the least useful information/item that you found on the Internet about stopping smoking? Why?
- 16. Given your experience, if a good friend wanted to stop smoking and they needed information, would you recommend they use the Internet? Why/Why not?

## Appendix 2

### Internet simulations

Prior to simulation:

Give the participant the opportunity to search generally on the Internet for information about stopping smoking.

Scenario 1

You have heard that Nicotine Replacement Therapy (NRT) doubles your chances of quitting. What types of NRT products are available? Are patches more effective than gum?

Scenario 2

You need help to stop smoking. What are the local support services available in your area? What are the contact details?

Scenario 3

You saw a report on television about Zyban®, a new drug to help people stop smoking. Are there any risks or side effects? Where can you buy it?
